# Gamma Knife Treatment of Brainstem Metastases

**DOI:** 10.3390/ijms15069748

**Published:** 2014-05-30

**Authors:** Halloran E. Peterson, Erik W. Larson, Robert K. Fairbanks, Alexander R. MacKay, Wayne T. Lamoreaux, Jason A. Call, Jonathan D. Carlson, Benjamin C. Ling, John J. Demakas, Barton S. Cooke, Ben Peressini, Christopher M. Lee

**Affiliations:** 1 Gamma Knife of Spokane, 910 W 5th Ave, Suite 102, Spokane, WA 99204, USA; E-Mails: hapeters@uw.edu (H.E.P.); elar5000@uw.edu (E.W.L.); Robert.fairbanks@ccnw.net (R.K.F.); gkspokane@armackay.net (A.R.M.); wayne.lamoreaux@ccnw.net (W.T.L.); jason.call@ccnw.net (J.A.C.); jcarlson@neuroandspine.com (J.D.C.); bling@neuroandspine.com (B.C.L.); jdemakas@rockwoodclinic.com (J.J.D.); bo@gkspokane.com (B.S.C.); 2 Cancer Care Northwest, 910 W 5th Ave, Suite 102, Spokane, WA 99204, USA; 3 School of Medicine, University of Washington, 1959 NE Pacific St, Seattle, WA 98185, USA; 4 MacKay & Meyer MDs, 711 S Cowley St, Suite 210, Spokane, WA 99202, USA; 5 Inland Neurosurgery & Spine Associates, 105 W 8th Ave, Suite 200, Spokane, WA 99204, USA; 6 Spokane Brain & Spine, 801 W 5th Ave, Suite 210, Spokane, WA 99204, USA; 7 DataWorks Northwest, LLC, Coeur D’Alene, ID 83814, USA; E-Mail: benperessini@dataworksnw.com

**Keywords:** brainstem metastases, Gamma Knife, radiosurgery

## Abstract

The management of brainstem metastases is challenging. Surgical treatment is usually not an option, and chemotherapy is of limited utility. Stereotactic radiosurgery has emerged as a promising palliative treatment modality in these cases. The goal of this study is to assess our single institution experience treating brainstem metastases with Gamma Knife radiosurgery (GKRS). This retrospective chart review studied 41 patients with brainstem metastases treated with GKRS. The most common primary tumors were lung, breast, renal cell carcinoma, and melanoma. Median age at initial treatment was 59 years. Nineteen (46%) of the patients received whole brain radiation therapy (WBRT) prior to or concurrent with GKRS treatment. Thirty (73%) of the patients had a single brainstem metastasis. The average GKRS dose was 17 Gy. Post-GKRS overall survival at six months was 42%, at 12 months was 22%, and at 24 months was 13%. Local tumor control was achieved in 91% of patients, and there was one patient who had a fatal brain hemorrhage after treatment. Karnofsky performance score (KPS) >80 and the absence of prior WBRT were predictors for improved survival on multivariate analysis (HR 0.60 (*p* = 0.02), and HR 0.28 (*p* = 0.02), respectively). GKRS was an effective treatment for brainstem metastases, with excellent local tumor control.

## 1. Introduction

Brain metastases are the most common intracranial malignancies, occurring in 15%–40% of adult cancer patients [1]. Among metastatic brain tumors, 3%–5% occur in the brainstem [2]. A study of 751 patients by Yen *et al.* found that the primary tumors with the highest rates of brainstem involvement were breast, ovarian, renal cell carcinoma, colorectal, lung, and melanoma [3]. The most common route of spread in these cases is hematogenous, though perineural spread has been documented [4]. The dense concentration of neural tracts and nuclei in the brainstem means that brainstem metastasis frequently cause significant neurological defects including cranial neuropathies, and motor and sensory deficits. Surgical resection of metastases in the brainstem is generally not an option. Chemotherapy is also of limited utility in these cases. Although relatively uncommon, brainstem metastases come with a poor prognosis; estimated survival without treatment is between one and six months [5].

Gamma Knife radiosurgery (GKRS) has become one of the primary tools for management of brainstem metastasis. Several retrospective case series have documented the value of GKRS in treating brainstem metastasis [2,3,5,6,7,8,9,10,11,12,13,14]. GKRS is minimally invasive, effective at sparing tissue that surrounds lesions, and is well tolerated by most patients allowing rapid return to pre-treatment activities. The palliative goal of GKRS is to control the growth of the brainstem metastasis and to prevent further neurological decline during their limited survival.

The aim of this study was to contribute to the growing body of literature demonstrating the safety and efficacy of radiosurgical treatment of brainstem metastasis.

## 2. Results and Discussion

### 2.1. Results

A total of 41 patients with brainstem metastases were analyzed (see Table 1). The median age at initial treatment was 59 years. Nineteen (46%) of the patients received whole brain radiation therapy (WBRT) prior to or concurrent with GKRS treatment. Thirty (73%) of the patients had a single brainstem metastasis at time of treatment. Multiple metastases throughout the central nervous system were treated concurrently with Gamma Knife. The average GKRS dose was 17 Gy (range 10–22.5 Gy). Average tumor volume was 0.66 cm^3^ (range 0.004–6.0 cm^3^).

**Table 1:** Patient population baseline characteristics.

Characteristic	Renal/Melanoma	SC Lung	Other Lung	Breast	Other/Unknown	Total
**Patient Number**	*n* = 7	*n* = 4	*n* = 17	*n* = 10	*n* = 3	*n* = 41
**Age at Diagnosis Mean/Median (Range) (years)**	69.3/73.0 (47–90)	60.0/60.5 (52–67)	59.1/59.0 (38–82)	56.2/52.5 (38–96)	64.7/66.0 (58–70)	60.6/59.0 (38–96)
<60	2	2	10	8	1	23
≥60	5	2	7	2	2	18
**Gender**						
Female	2	2	9	10	1	24
Male	5	2	8	0	2	17
**Prior WBRT**						
Yes	1	4	6	6	2	19
No	6	0	10	3	1	20
Unknown	0	0	1	1	0	2
**Lesion Number**						
1	6	3	11	7	3	30
>1	1	1	6	3	0	11
**Karnofsky Performance Score (KPS)**						
≤70	3	0	10	4	3	20
≥80	4	4	7	6	0	21
**Gamma Knife (GK) Dose (Gy)**						
<16	1	1	1	2	0	5
≥16	6	3	16	8	3	36
**Tumor Volume (cc)**						
<0.5	2	2	7	7	1	19
≥1.0	3	1	1	0	1	6
Unknown	2	1	2	10	1	16

For the entire population, the local tumor control rate was 91%. Local control was defined as no evidence of tumor progression at time of death or loss of follow-up. There was one patient in our series who died of a fatal brain hemorrhage on the day of his Gamma Knife procedure. This patient was 66 years old with metastatic melanoma, and was found to have a brainstem hemorrhage at the site of treatment. This was the only observed complication.

**Figure 1:**
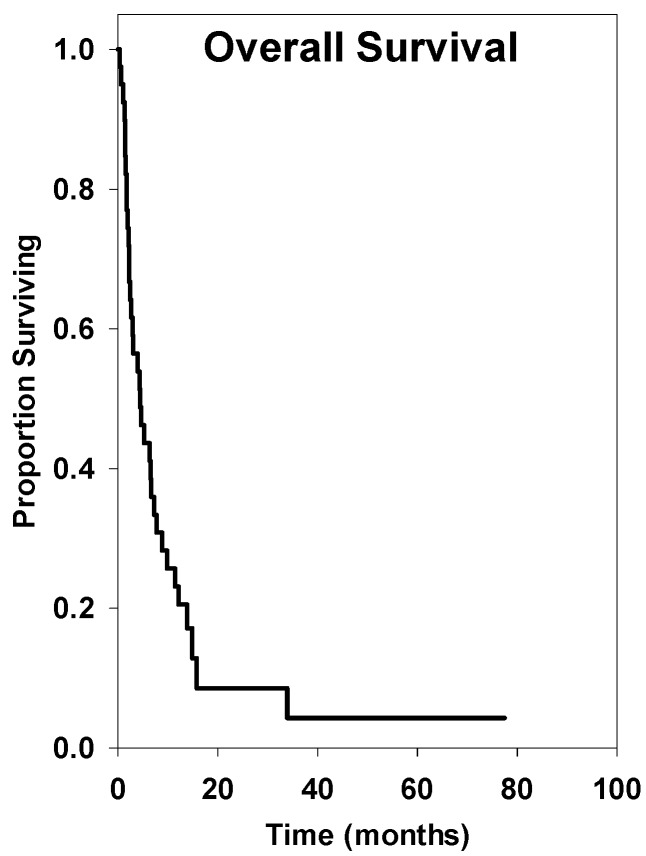
Overall survival of all patients with brainstem metastasis treated with Gamma Knife radiosurgery (GKRS) (*n* = 41).

The overall survival at 6 months was 42%, at 12 months was 22%, and at 24 months was 13% (see Figure 1). The median survival after treatment of brainstem metastasis was 4.40 ± 2.72 months. Nine patients were lost to follow-up.

**Figure 2:**
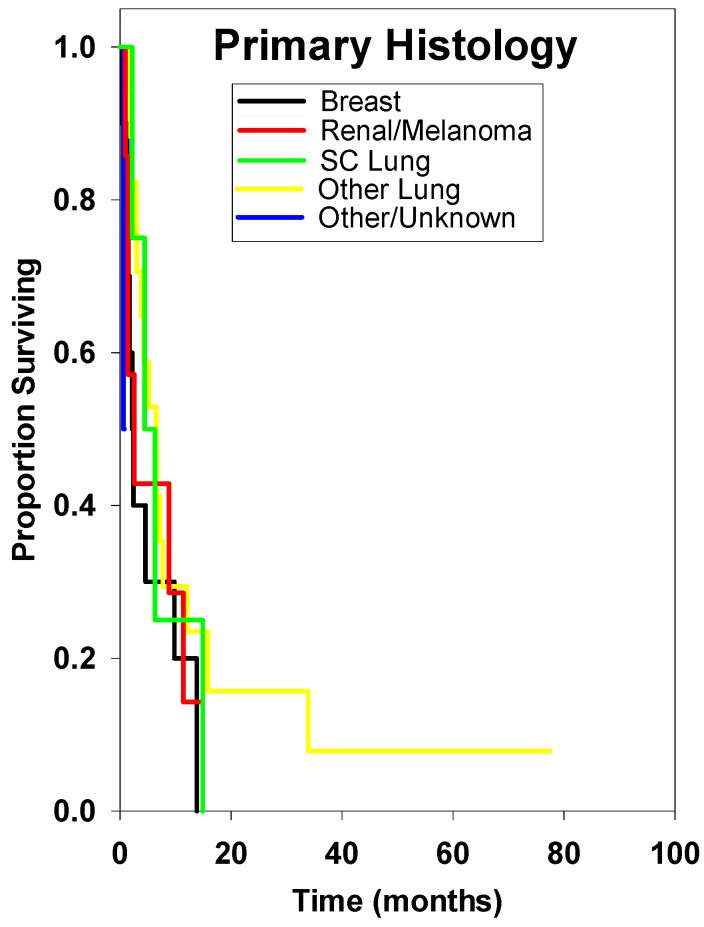
Survival curves subdivided by primary tumor histology. There was no statistical difference among these groups. See Table 1 for sample size.

**Figure 3:**
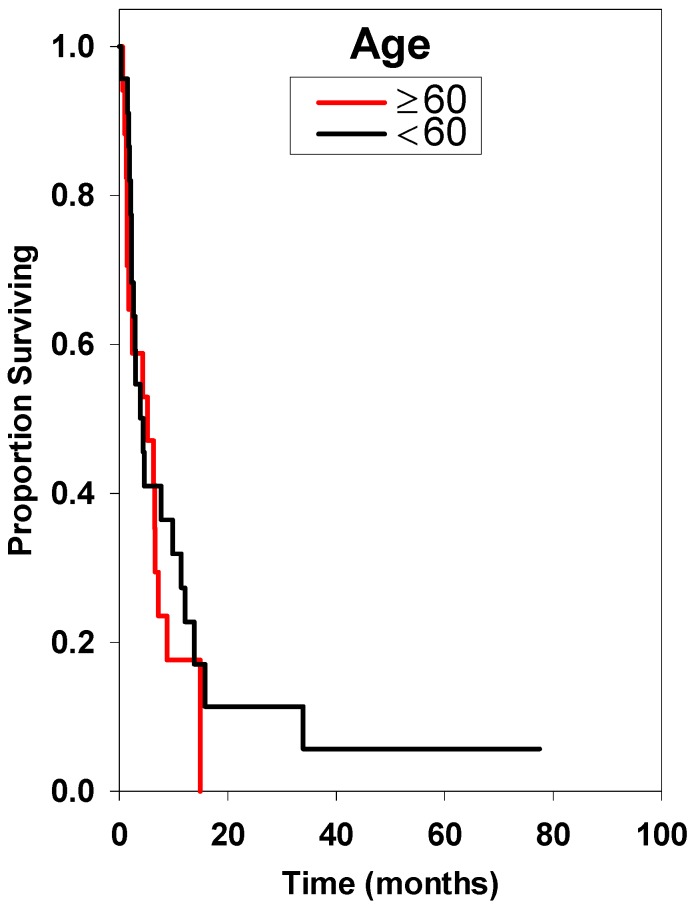
Survival curves based on age.

**Figure 4:**
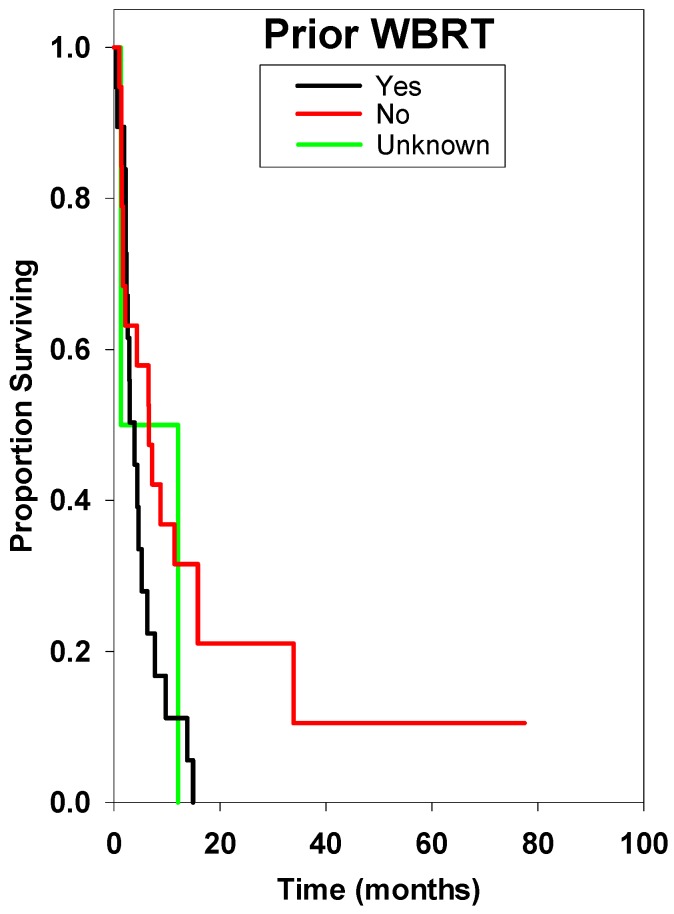
Survival curve comparing patients with whole brain radiation therapy (WBRT). There was a statistically significant reduced survival in patients with prior or concurrent WBRT in the multivariate analysis (*p* = 0.019). This finding was likely due to increased overall brain tumor load.

**Figure 5:**
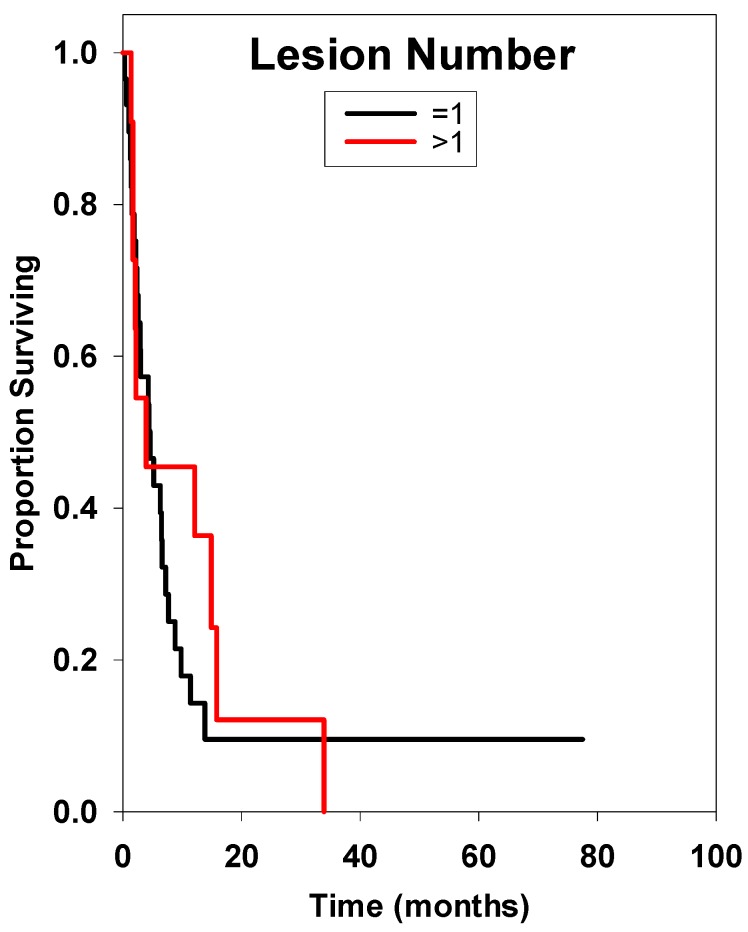
Survival curve considering the number of metastasis. There was no statistically significant difference between these two groups.

**Figure 6:**
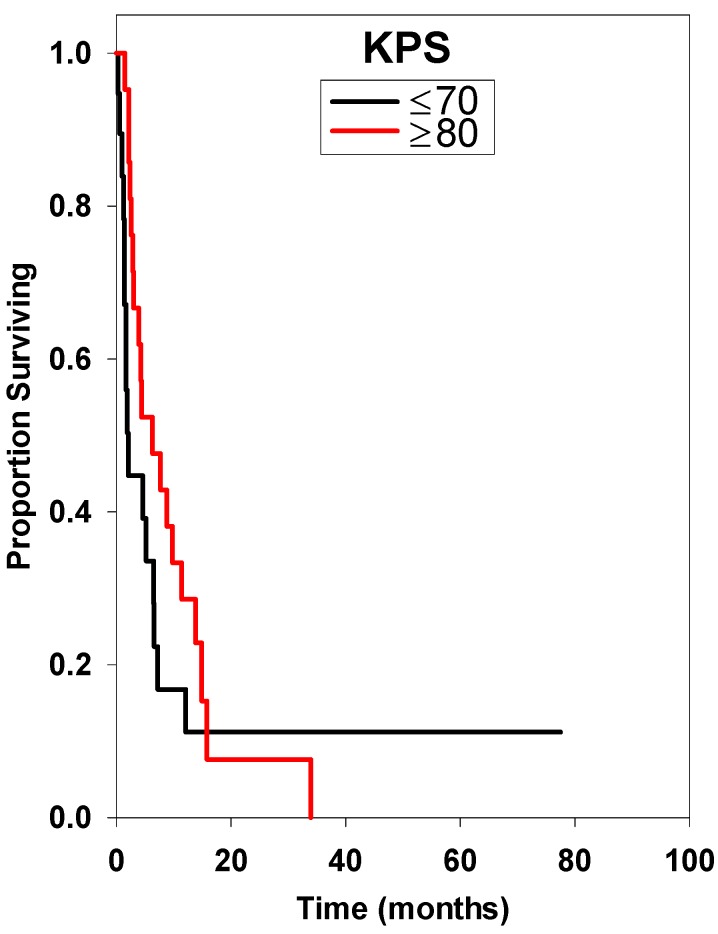
Survival curve considering Karnofsky performance score (KPS). There was a statistically significant difference with better survival in patients with KPS ≥ 80 (*p* = 0.019) after multivariate analysis.

**Figure 7:**
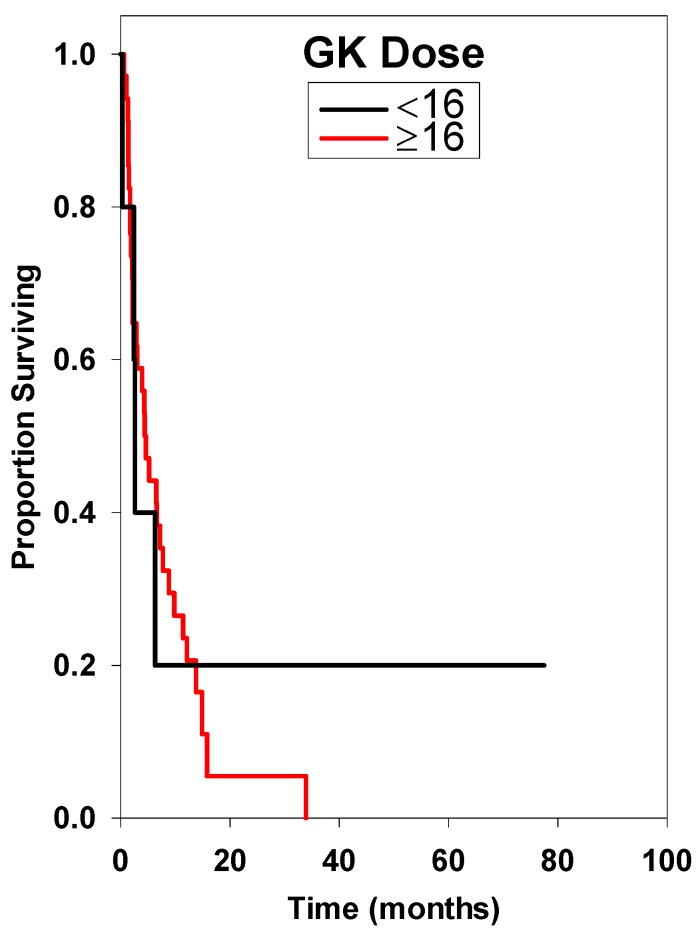
Survival curve comparing Gamma Knife (GK) radiotherapy dose or brainstem metastasis (<16 Gy *vs.* ≥16 Gy). There was no statistically significant difference among these groups.

**Figure 8:**
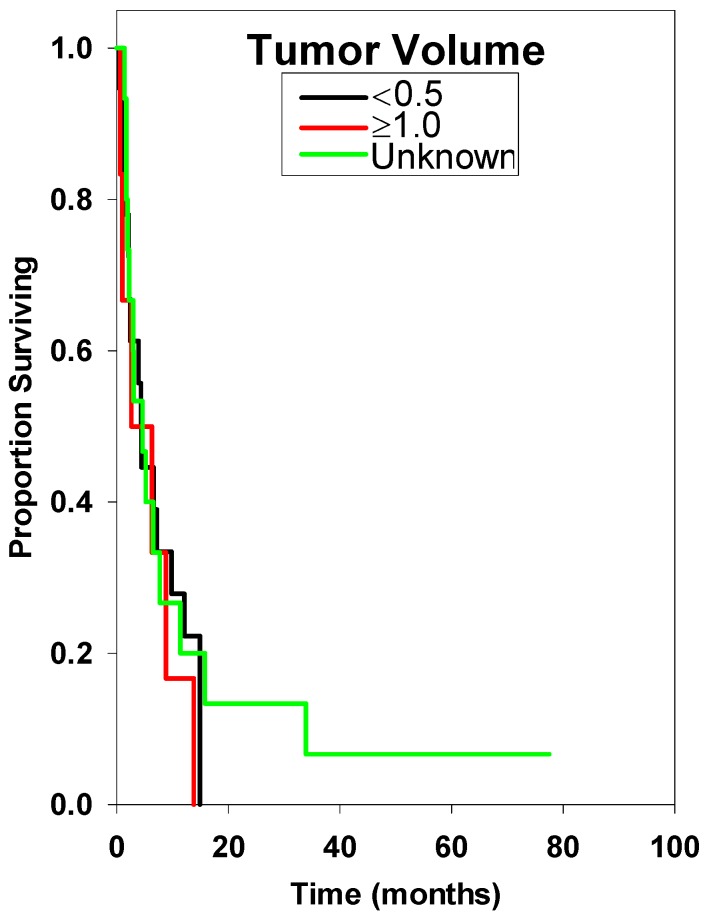
Survival curve comparing tumor volume (<0.5 cc *vs.* ≥1.0 cc). There was no statistically significant difference among these groups.

Univariate analysis of pretreatment clinical factors was performed. Factors potentially related to survival that were not found to be statistically significant were primary histology, age, lesion number, Gamma Knife dose, or tumor volume (Table 2, Figure 2, Figure 3, Figure 4, Figure 5, Figure 6, Figure 7, and Figure 8). Multivariate analysis was subsequently performed. This multivariate analysis found that Karnofsky performance score (KPS) ≥80 and the absence of prior whole brain radiation therapy were statistically significant predictors for improved overall survival (HR 0.60 (*p* = 0.02), and HR 0.28 (*p* = 0.02), respectively) (Table 3).

### 2.2. Discussion

Brainstem metastases, while relatively infrequent, are serious complications of cancer that indicate poor prognosis and can cause significant neurological disability impacting quality of life. To date, there has not been a randomized controlled trial of GKRS for brainstem metastases compared to palliative care alone or WBRT. Prior studies consist of fourteen retrospective case series published between 1999 and 2014 [2,3,5,6,7,8,9,10,11,12,13,14,15,16].

The emergence of GKRS as a treatment modality for managing brainstem metastasis calls for characterization of factors influencing outcomes. Identifying characteristics of patients who benefit most from GKRS has been the goal of many of these previously published case series. Favorable KPS has been consistently found to confer longer survival in previous studies [2,9,12]. Other measures of performance status found to be significant predictors of survival include Radiation Therapy Oncology Group—Recursive Partitioning Analysis (RTOG RPA) [15], and the Basic Score for Brain Metastases [12]. Control of systemic disease has also repeatedly been found to be a favorable prognostic factor for brainstem metastasis patients [3,6,9,12]. Given that systemic disease control and KPS performance have both been found so often to significantly predict survival, it stands to reason that RTOG RPA might in fact be the most useful prognostic scoring system for these patients. However, the retrospective design of all existing studies on the subject has likely limited investigators’ ability to collect complete data for calculating this parameter. Further factors associated with increased survival include smaller tumor volume [10,17] and single metastasis [8,9]. Metastatic melanoma was found to be associated with significantly worse outcomes in studies by Hatiboglu *et al.* and Kased *et al.* [8,17]. Kawabe *et al.* observed longer qualitative survival (maintaining a KPS >70) in patients who had higher KPS scores to begin and smaller tumor volume [9]. Since brainstem metastases are rarely the cause of death in these patients, qualitative survival post-GKRS should be a focus of future studies.

**Table 2:** Univariate analysis of median survival.

Characteristic	Median Survival		Hazard Ratio		
	*n*	95% CI	Estimate	95% CI	*p* Value **
**Histology**					
Breast *	10	2.40 ± 1.06	Reference		
SC Lung	4	4.40 ± 4.02	0.57	0.10–2.33	0.546
Other Lung	17	6.50 ± 3.10	0.57	0.23–1.54	0.235
Renal/Melanoma	7	2.60 ± 3.08	0.88	0.25–2.78	0.999
Other/Unknown	3	0.60 ± 0.96	8.33	0.44–504.80	0.097
**Age at Diagnosis (years)**					
<60 *	23	4.30 ± 5.82	Reference		
≥60	18	4.40 ± 1.94	1.37	0.65–2.88	0.379
**KPS**					
≤70 *	20	1.90 ± 0.74	Reference		
≥80	21	6.30 ± 5.08	0.60	0.29–1.23	0.155
**Lesion Number**					
1 *	30	4.40 ± 2.90	Reference		
>1	11	3.90 ± 10.79	0.72	0.30–1.59	0.468
**Gamma Knife (GK) Dose (Gy)**					
<16 *	5	2.60 ± 0.43	Reference		
≥16	36	4.40 ± 2.55	1.22	0.41–4.90	0.999
**Prior WBRT**					
Yes *	19	3.90 ± 2.05	Reference		
No	20	6.50 ± 6.57	0.55	0.25–1.194	0.101
Unknown	2	Incalculable	0.87	0.09–3.92	0.999
**Tumor Volume (cc)**					
<0.5 *	19	4.40 ± 1.03	Reference		
≥1.0	6	2.60 ± 6.36	1.45	0.45–4.00	0.428
Unknown	16	3.00 ± 3.33	1.00	0.43–2.27	0.999

* Reference group against which other groups’ survival experience are compared; ** *p* value for log-rank testing the null hypothesis that the groups’ survival experience is same as reference group.

**Table 3:** Multivariate analysis of median survival. Prior whole brain radiation therapy and Karnofsky performance score (KPS) were statistically significant predictors of survival.

Characteristic	Hazard Ratio		
	Estimate	95% CI	*p* Value **
**Histology**			
Breast *	Reference		
Small Cell Lung	0.51	0.10–2.69	0.427
Other Lung	0.54	0.19–1.53	0.242
Renal/Melanoma	2.10	0.50–8.82	0.310
Other/Unknown	5.31	0.30–4.03	0.255
**Age at Diagnosis (years)**			
<60 *	Reference		
≥60	1.39	0.55–3.53	0.493
**KPS**			
<70 *	Reference		
≥80	0.31	0.12–0.82	0.019
**Lesion Number**			
1 *	Reference		
>1	1.43	0.50–4.12	0.507
**Gamma Knife (GK) Dose (Gy)**			
<16 *	Reference		
≥16	1.30	0.38–4.45	0.676
**Prior WBRT**			
Yes *	Reference		
No	0.28	0.10–0.81	0.019
Unknown	0.38	0.05–2.82	0.341

* Reference group against which other groups’ survival experience are compared; ** *p* value for test if groups’ survival experience is same as reference group.

Adverse effects of Gamma Knife (GK) treatment remain an important consideration in these cases. Hong *et al.* analyzed 279 radiosurgery procedures for brain metastases and found that 34% of patients experienced acute, mild to moderate sequelae including headache, seizures, and fluid retention while less than 2% experienced serious adverse effects requiring hospitalization [18]. The average rate of adverse effects reported in previous studies of brainstem metastases is 6% (range 0%–27%) [2,3,5,6,7,8,9,10,11,12,13,14]. This low number may be the result of varied reporting methods among the studies with some reporting only serious events, or it could be that in a short-surviving population such as brainstem metastasis patients, late-arising complications are masked.

Kilburn *et al.* sought to define volume toxicity thresholds for GKRS treatment of brainstem metastasis and found a significant correlation between increasing tumor volume and increased hazard for toxicity [16]. Patients in this series with tumor volume greater than 1 cm^3^ had significantly higher rates of treatment related complications at 6 months and 1 year [16]. Kased *et al.* found the same increased risk of toxicity as well as lower overall survival among patients with tumors greater than 1 cm^3^ [8]. Of note our study did not identify a relationship between tumor volume and survival. While the threshold of 1 cc chosen by Kilburn *et al.* warrants further investigation, it is based on only 4 toxicity events that occurred in a study of 44 patients [16]. It will be important that future studies aim to discover optimal thresholds that limit adverse events without sacrificing local control.

Currently, there are no dosage guidelines for the treatment of brainstem metastases, so dosing in previous studies tended to be based either on conservative estimates or prior work by other investigators. Previous studies have used doses ranging from 13 to 20 Gy and achieved average local control of 89% (range 77%–100%) [2,3,5,6,7,8,9,10,11,12,13,14,15,16]. The long-standing belief that the brainstem is more radiosensitive than the cerebrum seems to be largely based on work done by Boden *et al.* in 1948 [19]. However, it may be noted that among the existing studies of GKRS for brainstem metastases patients, the two with the highest average marginal dose, Lorenzoni *et al.* and Fuentes *et al.* using 20 and 19.6 Gy to treat 25 and 28 patients respectively each reported zero complications [5,12]. The effect of radiation dose in terms of patient benefits is unclear. Among existing studies, Valery *et al.* used one of the lowest average doses in their series with 13.4 Gy and achieved 90% local tumor control and a median survival time (MST) of 10 months [20]. These results are similar to those found by Lorenzoni *et al.* who with an average marginal dose of 20 Gy observed local control of 95% and MST of 11.1 months.

In nearly all published studies on GKRS for brainstem metastases, WBRT is used prior to or concurrent with treatment in a portion of patients [3,5,6,7,8,9,10,12,13,14,15,16]. Lorenzoni *et al.* found as we did that patients who did not receive WBRT survived longer than those who did. These findings are likely due to selection bias. Patients in the current series who received WBRT tended to be those who had multiple brainstem metastases or had extensive intracranial disease. Beyond these findings, there are no other data specifically regarding the effect of combination therapy on brainstem metastases. However, data inclusive of all intracranial metastases is growing. A review by Patil *et al.* on treatment of brain metastases shows that WBRT plus stereotactic radiosurgery (SRS) yielded better local tumor control (HR 0.27; 95% CI 0.14 to 0.52) and improved KPS performance status but is not associated with longer survival [21]. Aoyama *et al.* found reduced recurrence of distant intracranial tumors requiring salvage among patients receiving combination therapy, but did not find a difference in survival times [22]. Recent studies have shown that the addition of WBRT may be detrimental in terms of neurocognitive function and support an upfront approach of SRS therapy alone. Chang *et al.* found significantly increased risk of decline in memory and learning abilities in patients who had WBRT, and Soffietti *et al.* found a decreased quality of life using the Health Related Quality of Life (HRQOL) inventory [23,24].

For patients with solitary or limited brainstem metastases, our current approach is upfront SRS of at least 16 Gy depending on tumor size and histology. WBRT is used for later salvage if these cases progress to more disseminated intracranial disease. When patients present with multiple brain metastases, we usually employ WBRT to 3750 cGy with an SRS boost to 16 Gy or less.

The primary limitation of the present study is its retrospective design. Additionally, while it is among the larger existing studies of brainstem metastases patients, it is limited by a relatively small sample size.

## 3. Experimental Section

In this retrospective chart review we examined a sequential group of patients treated with Gamma Knife with brainstem metastases between 2005 and 2013. This research was approved by the Institutional Review Board—Spokane in March 2013. Patient lists were generated from a detailed database of all lesions treated with GKRS. For the analysis the patients were grouped by primary histology (renal cell and melanoma, small cell lung, other lung, breast, other and unknown), by age at diagnosis (<60 and ≥60 years), by prior whole-brain radiation therapy (WBRT) (yes, no and unknown), by number of lesions (1 and >1), by KPS value (<70 and ≥80), Gamma Knife treatment dose (<16 and ≥16 Gy), and by tumor volume (<0.5 and ≥1 cm^3^).

Patients were treated at Gamma Knife of Spokane using the Model C Leksell ^60^Co Gamma Knife (Elekta, Stockholm, Sweden). Prior to the GKRS procedure, local anesthetic was applied to facilitate placement of the stereotactic head frame. Gadolinium enhanced magnetic resonance imaging of the head within the coordinate frame was performed and then a neurosurgeon, radiation oncologist, and medical physicist concurrently planned the radiosurgery treatment. Follow-up MRI was performed 1 month post-GRKS and then every 2 months after that. Any patient still living that had not been contacted within 6 months was deemed lost to follow-up.

Survival curves were estimated using the Kaplan-Meier method and used to compare primary histology groups, age groups, prior WBRT groups, lesion number groups, KPS groups, and gamma knife treatment dose groups. Andersen 95% confidence intervals for the median survival time of the groups were calculated. Approximate confidence intervals for the log hazard-ratio were calculated. Log-rank tests were employed to determine if there is statistical evidence of differences between the survival curves of the groups. Finally, the Cox proportional hazard model was used in a multivariate analysis of the primary histology groups, age groups, prior WBRT groups, lesion number groups, KPS groups, and Gamma Knife treatment dose groups. All statistical analyses utilized StatsDirect Version 2.7.3 (StatsDirect Ltd., Altrincham, UK) and/or SigmaPlot Version 12.0 (SYSTAT Software, Inc., San Jose, CA, USA).

## 4. Conclusions

Metastases occurring in the brainstem are rare, have limited treatment options, and portend a poor prognosis. In this series, GKRS was shown to be a safe treatment modality for brainstem metastases and provides excellent local tumor control. Although Gamma Knife is a feasible treatment option for patients with brainstem metastases, much research is still needed in the future to improve clinical outcomes for this challenging oncologic scenario.
